# Network meta-analysis of clinical trials of fluid treatments in sepsis demonstrates improved survival with albumin compared with crystalloid and hydroxyethyl starch

**DOI:** 10.1186/cc12313

**Published:** 2013-03-19

**Authors:** M Bansal, A Farrugia, G Martin

**Affiliations:** 1 Plasma Protein Therapeutics Association, Annapolis, MD, USA; 2 University of Western Australia, Crawley, Australia; 3 3Emory University School of Medicine, Atlanta, GA, USA

## Introduction

Fluid resuscitation is widely practiced in treating sepsis. Comparative assessment of the different fluid modalities is hampered by a paucity of direct trials. We present a network meta-analysis for assessing the relative effectiveness of two fluid treatments in sepsis when they have not been compared directly in a randomized trial but have each been compared with a common treatment.

## Methods

A systematic review of trials sepsis yielded 13 trials for assessment in network meta-analysis. The indirect comparison between albumin, HES and crystalloid was conducted using Bayesian methods for binomial likelihood, fixed-effects network meta-analysis with a Monte Carlo Gibbs sampling method. Studies in septic patients with crystalloid as a reference treatment compared with any formulation of the colloid treatments albumin or HES were included, as were direct head-to-head trials between the two colloids.

## Results

**Figure 1:**
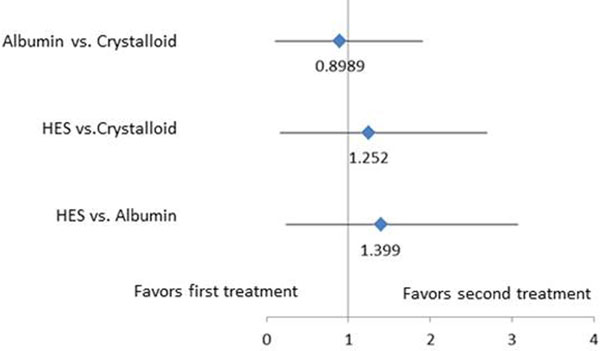
**Forest plot of results of Bayesian fixed-effect network meta-analysis of mortality**.

Odds ratios between the different treatments were obtained (Figure 1). Ranking the interventions [1] demonstrated that albumin ranked highest in lowering mortality at a 96.4% probability compared with 3.6% and 0.01% for crystalloid and HES, respectively.

## Conclusion

Albumin as a fluid therapy in sepsis is associated with the lowest mortality of the three modalities studied.
